# Performance Optimization in Frequency Estimation of Noisy Signals: Ds-IpDTFT Estimator

**DOI:** 10.3390/s23177461

**Published:** 2023-08-28

**Authors:** Miaomiao Wei, Yongsheng Zhu, Jun Sun, Xiangyang Lu, Xiaomin Mu, Juncai Xu

**Affiliations:** 1 Department of Electronic and Information, Zhongyuan University of Technology, Zhengzhou 450007, China; zhuys@zut.edu.cn (Y.Z.); sunjun@zut.edu.cn (J.S.); luxy@zut.edu.cn (X.L.); 2 Department of Information Engineering, Zhengzhou University, Zhengzhou 450001, China; iexmmu@zzu.edu.cn; 3 Department of Civil Engineering, Case Western Reserve University, Cleveland, OH 44106, USA

**Keywords:** Ds-IpDTFT, fine frequency estimation, noisy exponential signals, computational complexity, signal processing

## Abstract

This research presents a comprehensive study of the dichotomous search iterative parabolic discrete time Fourier transform (Ds-IpDTFT) estimator, a novel approach for fine frequency estimation in noisy exponential signals. The proposed estimator leverages a dichotomous search process before iterative interpolation estimation, which significantly reduces computational complexity while maintaining high estimation accuracy. An in-depth exploration of the relationship between the optimal parameter *p* and the unknown parameter δ forms the backbone of the methodology. Through extensive simulations and real-world experiments, the Ds-IpDTFT estimator exhibits superior performance relative to other established estimators, demonstrating robustness in noisy conditions and stability across varying frequencies. This efficient and accurate estimation method is a significant contribution to the field of signal processing and offers promising potential for practical applications.

## 1. Introduction

The problem of precise and efficient frequency estimation in noisy signals, essential for signal synchronization, is pervasive in numerous domains such as power transmission [1,2,3], satellite control [4,5], mechanical systems [6,7], mobile communication [8,9], radar systems [10,11,12], and biomedical signal processing [13]. To address this issue, a multitude of strategies have been proposed. Notably, estimators informed by the periodogram maximizer [14] estimate the frequency of a time-domain signal via the maximum search of the Discrete Fourier Transform (DFT) spectrum, leveraging the time-frequency characteristics of the received signals for an easy-to-achieve frequency estimation [15].

However, the accuracy of these estimators is often limited by the frequency resolution of DFT. Because the signal sampling period is usually asynchronous with its real period, the energy of the signal leaks into adjacent frequency bins, producing an estimation bias, namely spectral leakage. In this case, the estimation error is limited to less than one frequency resolution without noise interference. In response, several interpolation techniques have been introduced. These include methods like Quinn [16,17], Macleod [18], Jacobsen [19], Yang [20], Candan [21,22], parabolic [23,24], and Fang [25], which utilize assistant DFT samples. By analyzing the relationship between the frequency offset to be estimated and the adjacent DFT samples, the interpolation formula is obtained, and the estimation error of these estimators can be reduced to less than half a frequency resolution through several multiplications, which is very useful for practical applications with limited computing resources. The mean square error (MSE) of parabolic [24] can reach 1.0033 times the Cramér–Rao lower bound (CRLB). The utilization of these interpolation techniques, in addition to an alternative strategy that employs signal samples with zero padding for DFT calculation [20], has shown that iterative operations are beneficial for error reduction, because padding zeros after signal samples is equivalent to reducing the range of the frequency offset to be estimated, and the iterative operation helps to reduce the dependence of the interpolation formula on actual signal frequency. The methods can be used for frequency estimation with limited signal samples and strong noise, such as burst communication and satellite communication. This is evident in [26], where the root mean square error (RMSE) of the estimator reached 1.014 times the ACRB after two iterations. The use of windows for signal filtering while computing DFT can further enhance estimation accuracy [3,27], and various windows, such as the quasi-synchronous window [28], combined cosine window [29], and triangular self-convolution window [30], have been designed to decrease estimation error caused by spectral leakage. The suppression of spectral leakage is realized by performing windows on the signal samples to reduce the discontinuity introduced by the truncation of signal sequences, which is useful for spectral leakage and harmonic interference in practice. However, these improved methods require substantial matrix computations, rendering them computationally costly.

Despite these improvements, the accuracy of interpolation estimators is still influenced by the frequency resolution Δf, which is the ratio of the discrete sampling frequency to the discrete sampling number. In response, many interpolations using discrete time Fourier transform (DTFT) samples, which are closer to the DFT maximum than DFT samples, have been explored [31,32]. Without considering noise, the estimation range of the frequency offset can be reduced to less than a quarter of the frequency resolution by using DTFT samples 0.5Δf away from the spectral maximum for interpolation. This technique has demonstrated a further reduction in the estimation error after two iterations. It is suitable for application scenarios that requires fast and accurate communication, such as high-speed trains and aircraft. The estimators proposed by Fan [33] and Serbes [34], which achieved state-of-the-art performance by integrating most optimization methods described above, can still be optimized for stability.

In the pursuit of further improvements, we propose a new estimator, named Ds-IpDTFT, derived from dichotomous search and iterative interpolation on DTFT samples. The estimator includes two stages: the first stage provides a coarse frequency estimate by locating the maximum of the DFT spectrum, followed by a second stage that employs a dichotomous search to narrow the estimation range. Afterwards, the fine frequency estimate is iteratively calculated according to an asymptotically unbiased closed-form formula. This proposed method integrates many of the above-discussed techniques, aiming to improve not only estimation accuracy but also computational efficiency and stability. By introducing the dichotomous search into the frequency estimation, the estimation error is expected to reach less than one eighth of the frequency resolution after dichotomous search, and the computation is slightly reduced, and explained in Section 4. Thus, it provides an effective method to realize fast and accurate communication in high dynamic conditions.

The main contributions of this study are as follows:

- The study introduces the Ds-IpDTFT estimator, a pioneering methodology for fine frequency estimation in noisy exponential signals.
- A comprehensive theoretical analysis is provided, with a focus on the relationship between the parameters *p* and δ.
- The performance of the Ds-IpDTFT estimator is validated through rigorous simulations and real-world experiments.
- The research propels the field forward by presenting a more accurate and efficient estimator, thereby opening up new pathways for future research.

The remainder of the paper is organized as follows. Section 2 discusses the signal model with additive noise, the CRLB expression, and a generalized iterative interpolation estimator. In Section 3, a comprehensive discussion on the principle of the new estimator ensues, with detailed steps of the proposed estimator. This leads into Section 4, where we analyze the performance of the Ds-IpDTFT compared with other estimators and CRLB through simulations. Finally, in Section 5, we verify the proposed estimator on real data and conclude our work in Section 6.

## 2. Preliminaries

In this section, we consider a discrete-time exponential signal transmitted over the AWGN channel. The model of this signal in noise can be described as in Equation (1):

(1) $$\begin{array}{cc} y(n) & =x(n)+z(n)\\ \; & =Ae^{j(2\pi \frac{f}{f_{s}}n+\phi_{0})}+z\left(n\right), \end{array}$$

where $x\left(n\right)=A\exp\left(j\left(2\pi \frac{f}{f_{s}}n+\phi_{0}\right)\right)$ represents the discrete-time complex exponential signal; $\phi_{0}$ and *A* represent the phase and magnitude of the signal, respectively; $f/f_{s}$ represents the normalized frequency to be estimated, which is divided by the discrete sampling frequency $f_{s}$; n=0,1,…,N−1 represents the index of the received signal samples; and *N* represents the length of the received signal sequence. z(n), on the other hand, follows the distribution $N(0,\sigma^{2})$ and indicates complex additive Gaussian noise. As a result, the normalized frequency can be denoted as in Equation (2):

(2) $$\frac{f}{f_{s}}=\frac{k_{m}+\delta }{N},$$

where $k_{m}$ represents the integer part of the normalized frequency and equals the index of the DFT maximum obtained in the first estimation stage. Furthermore, −0.5<δ<0.5 represents the fractional part of the normalized frequency, which is computed in the second estimation stage.

### 2.1. CRLB of Frequency Estimation

Transitioning to the topic of frequency estimation, we note that the CRLB places a low bound on the MSE of the estimate, as presented in Equation (3) [14]. Here, γ denotes the signal-to-noise ratio (SNR), which can be computed by $\gamma =\frac{A^{2}}{\sigma^{2}}$.

(3) $$CRLB=\frac{3}{2\pi^{2}\gamma \cdot N\left(N^{2}-1\right)}.$$

### 2.2. Generalized Iterative Interpolation Estimator

In the context of interpolation estimators, a generalized iterative interpolation using magnitudes of optional DTFT samples has been proposed in [35]. This estimator is characterized by its flexible form and is known to obtain an estimation accuracy comparable with other state-of-the-art estimators. In this scheme, the coarse estimate is computed by locating the position of the DFT maximum, followed by estimating the residual frequency offset through iterative interpolations on the DFT maximum, $X(k_{m})$, and two neighboring DTFT samples, $X(k_{m}\pm p)$, next to the DFT maximum. Their expressions can be described as in Equation (4).

(4) $$X\left(k_{m}\pm p\right)=\sum_{n=0}^{N-1}Ae^{j\left[2\pi \left(\frac{f}{f_{s}}-\frac{k}{M}\right)n+\phi_{0}\right]}{|}_{f=\left(k_{m}+\delta \right)\Delta f,k=k_{m}\mp p}.$$

In the above equation, *p* denotes the distance between the index of the DFT maximum, $k_{m}$, and the index of the neighboring DTFT samples, $k_{m}\pm p$. *M* denotes the number of data used in the DFT computation, and M=2N signifies that *N* zeros are padded after *N* signal samples. Lastly, Δf represents the frequency resolution of DFT.

The expression of $X(k_{m}\pm p)$ can be simplified to Equation (5), and the matched magnitudes of $X(k_{m}\pm p)$ are given in Equation (6).

(5) $$X\left(k_{m}\pm p\right)=Ae^{j\phi_{0}}e^{j\pi \frac{N-1}{M}\left(\delta \mp p\right)}\frac{\sin(\pi (\delta \mp p))}{\sin(\frac{\pi }{M}\left(\delta \mp p\right))}.$$

(6) $$\left|X(k_{m}\pm p)\right|=A\left|\frac{\sin(\frac{\pi N}{M}\left(\delta \mp p\right))}{\sin(\frac{\pi }{M}\left(\delta \mp p\right))}\right|.$$

In order to further simplify the expression, let $X(k_{m}-p)$, $X(k_{m})$, and $X(k_{m}+p)$ be represented as $X_{k_{m}-p}$, $X_{k_{m}}$, and $X_{k_{m}+p}$, respectively. Considering that M/N>2 and $\left|\delta \mp p\right|\leq 1$, the ratio between $\sin(\frac{\pi N}{M}\left(\delta \mp p\right))$ and $\sin(\frac{\pi }{M}\left(\delta \mp p\right))$ is positive, hence the expression of $\left|X(k_{m}\pm p)\right|$ becomes as in Equation (7).

(7) $$\left|X_{k_{m}\pm p}\right|=A\frac{\sin(\frac{\pi N}{M}\left(\delta \mp p\right))}{\sin(\frac{\pi }{M}\left(\delta \mp p\right))}.$$

Subsequently, the ratio between $\left|X_{k_{m}\pm p}\right|$ and $\left|X_{k_{m}}\right|$ can be written as in Equation (8).

(8) $$\frac{\left|X_{k_{m}\pm p}\right|}{\left|X_{k_{m}}\right|}=\frac{\sin(\frac{\pi N}{M}\left(\delta \mp p\right))}{\sin(\frac{\pi }{M}\left(\delta \mp p\right))}/\frac{\sin(\frac{\pi N}{M}\delta )}{\sin(\frac{\pi }{M}\delta )}.$$

Assuming that M≫π(δ∓p), Equation (8) approximates to equation

(9) $$\begin{array}{c} \frac{\left|X_{k_{m}\pm p}\right|}{\left|X_{k_{m}}\right|}\approx \frac{\sin(\frac{\pi N}{M}\left(\delta \mp p\right))}{\sin(\frac{\pi N}{M}\left(\delta \right))}/\frac{\delta \mp p}{\delta }. \end{array}$$

Following some transformations, we obtain the sum as expressed in Equation (10).

(10) $$\frac{\left|X_{k_{m}+p}\right|}{\left|X_{k_{m}}\right|}\frac{\delta -p}{\delta }+\frac{\left|X_{k_{m}-p}\right|}{\left|X_{k_{m}}\right|}\frac{\delta +p}{\delta }\approx 2\cos\left(\frac{\pi N}{M}p\right).$$

Finally, the estimate $\hat{\delta }$ is computed by Equation (11). This generalized iterative interpolation estimator’s flexible form is clearly shown in the equation and can be regarded as a generalization of some iterative estimators with an equivalent interpolation equation and a determined value of *p*.

(11) $$\hat{\delta }=\frac{p\left(\left|X_{k_{m}+p}\right|-\left|X_{k_{m}-p}\right|\right)}{\left|X_{k_{m}+p}\right|+\left|X_{k_{m}-p}\right|-2\left|X_{k_{m}}\right|\cos\left(\frac{\pi N}{M}p\right)}.$$

The corresponding mean square error (MSE) of the generalized interpolation estimator can be expressed as in Equation (12) (the detailed deduction process is available in [35]).

(12) $$\begin{array}{c} E\left[{\left(\hat{\delta }-\delta \right)}^{2}\right]\approx \left\{\begin{array}{c} \begin{array}{c} \frac{\pi^{2}N}{M^{2}}\cdot \frac{\delta^{2}{\left(\left(\delta^{2}-p^{2}\right)\right)}^{2}}{\gamma }\cdot \\ \frac{p^{2}+\delta^{2}+2\delta^{2}\cos^{2}\left(\frac{\pi N}{M}p\right)-\left(3\delta^{2}+p^{2}\right)\mathrm{sinc}\left(\frac{2\pi N}{M}p\right)}{4p^{2}{\left(p\sin\left(\frac{\pi N}{M}\delta \right)\cos\left(\frac{\pi N}{M}p\right)-\delta \sin\left(\frac{\pi N}{M}p\right)\cos\left(\frac{\pi N}{M}\delta \right)\right)}^{2}},\\ \delta \neq 0\;\&\;\left|\delta \right|\neq p \end{array}\\ \frac{p^{2}}{4N\cdot \gamma }\cdot \frac{1-\mathrm{sinc}\frac{2\pi N}{M}p}{{\left(\mathrm{sinc}\frac{\pi N}{M}p-\cos\left(\frac{\pi N}{M}p\right)\right)}^{2}},\delta =0\\ \frac{2p^{2}}{N\cdot \gamma }\cdot \frac{1+\cos^{2}\left(\frac{\pi N}{M}p\right)-2\mathrm{sinc}\frac{2\pi N}{M}p}{{\left(1-\mathrm{sinc}\frac{2\pi N}{M}p\right)}^{2}},\left|\delta \right|=p \end{array}\right. \end{array}.$$

## 3. Proposed Estimator

**Figure 1:**
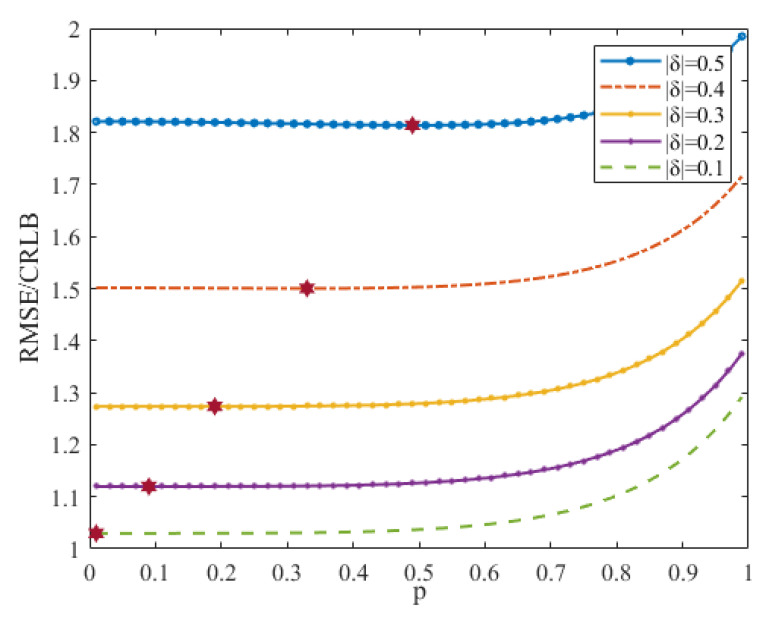
RMSE/CRLBs of the generalized iterative interpolation estimator versus *p* for various |δ|.

This section introduces a novel estimator inspired by the correlation between the offset *p* and the residual frequency offset that requires estimation. To observe this correlation, a simulation is carried out, and the results are visualized in Figure 1.

From Figure 1, the minimal root mean square errors (RMSEs) of the generalized iterative interpolation estimator for different $\left|\delta \right|$ at γ=0 dB and N=512 are labeled as a red hexagram. These RMSEs show variation with *p*, suggesting that an appropriate choice of *p* can minimize the estimation error of δ. However, due to its correlation with the unknown parameter δ, *p* cannot be precisely determined. Therefore, we narrow down the selection range of *p* using the dichotomous search algorithm, an efficient method for extreme search in a limited range, to reduce the estimation error of the generalized iterative interpolation estimator. The fine frequency estimation is initiated with the dichotomous search, allowing us to define a narrowed range of *p* before proceeding with interpolation.

In the dichotomous search, which includes two search steps, we initially compare the left and right DFT samples ($X_{k_{m}-1}$ and $X_{k_{m}+1}$) surrounding the DFT maximum. Depending on the results, we establish the next search range. If $X_{k_{m}+1}$ exceeds $X_{k_{m}-1}$, then the range is $\left(k_{m},k_{m}+0.5\right)$ as $X_{k_{m}}$ is larger than $X_{k_{m}+1}$, based on the outcome of the coarse frequency estimation. On the contrary, if $X_{k_{m}-1}$ is larger than $X_{k_{m}+1}$, the range is $\left(k_{m}-0.5,k_{m}\right)$, since $X_{k_{m}}$ exceeds $X_{k_{m}-1}$. For the subsequent search, we compute the DTFT samples $X_{k_{m}+0.5}$ or $X_{k_{m}-0.5}$ as the edge point of the next range. Continuing this process further refines the search range. After the dichotomous search is complete, the final estimation of the residual frequency offset is carried out using the generalized iterative interpolation estimator.

For the next search range, suppose it is $\left(k_{m},k_{m}+0.25\right)$. In the first iteration, the interpolation of the generalized estimator is conducted on $X_{k_{m}}$, $X_{k_{m}+0.125}$, and $X_{k_{m}+0.25}$ with p=0.125. Subsequent iterations follow the same methodology as outlined in Algorithm 1.

The process outlined in Algorithm 1 concludes that only one-bin DTFT needs to be computed during one iteration of the dichotomous search, while the generalized iterative interpolation estimator may need zero or three one-bin DTFT computations depending on whether the iteration parameter *q* equals one or not.
**Algorithm 1:** Proposed dichotomous search-enhanced IpDTFT (Ds-IpDTFT) estimator
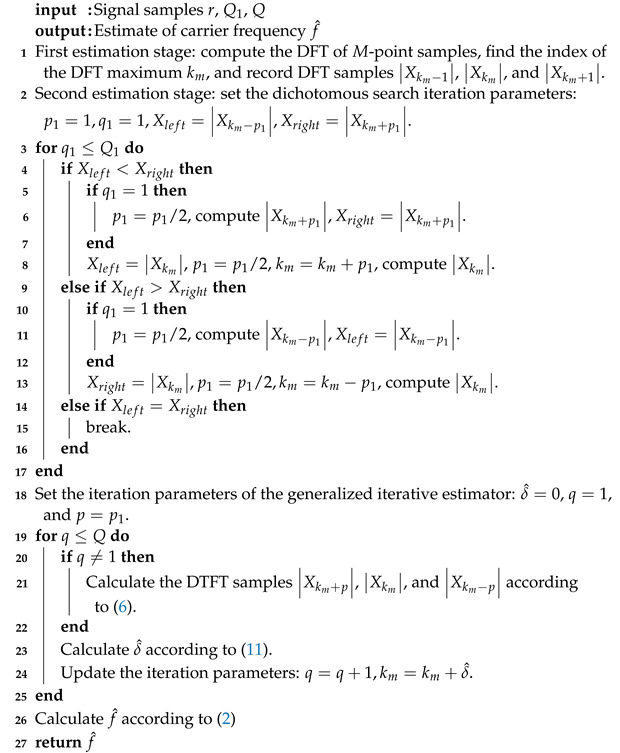


### 3.1. Discussion of M and N

**Figure 2:**
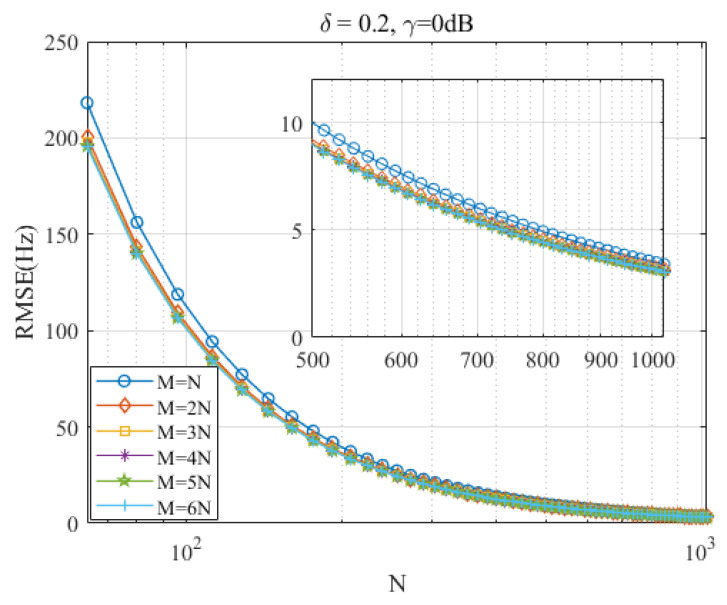
RMSEs of the generalized iterative interpolation estimator versus *N* for different *M*.

The data length, *M*, and signal length, *N*, are two crucial parameters of the generalized iterative interpolation estimator. Their impact on estimation accuracy is observed by plotting the RMSE curves of the generalized iterative interpolation estimator versus *N* for different *M* at γ=0 dB. This is visualized in Figure 2, where *N* varies from 16 to 1024 in steps of 16.

It can be inferred from Figure 2 that the RMSEs of the generalized iterative interpolation estimator decrease as *N* increases, with the estimator’s RMSEs falling below 10 Hz when *N* exceeds 500. However, the improvement of the RMSE diminishes with increasing *N*. The RMSEs can also be reduced by increasing *M*, but, when *M* exceeds 2N, the RMSE’s improvement is limited. Increasing the data length, *M*, and signal length, *N*, have similar effects, but they work on different principles. As the signal length, *N*, increases, the frequency resolution decreases, but the estimation accuracy is improved. With the increasing of the data length, *M*, the range of frequency offset to be estimated is reduced, but the frequency resolution remains the same. Although larger *M* or *N* values lead to better estimates, they require higher computational complexity due to calculations of DFT and DTFT.

### 3.2. Discussion of Q and $Q_{1}$

The parameters *Q* and $Q_{1}$, representing the iteration numbers of the generalized iterative interpolation estimator and the dichotomous search, respectively, are crucial in the proposed estimator. While a larger $Q_{1}$ can further narrow the search range, it requires more DTFT computation. For every increase of one of $Q_{1}$, the search range of the frequency offset is halved, but one DTFT computation is added. *Q* exhibits a similar effect. For every increase of one of *Q*, the estimation error is reduced, but the DTFT computation is doubled. Detailed discussions about *Q* can be found in [33,34], which recommend a value of two because the improvement in estimation error is very limited for Q≥3. However, considering the interaction between *Q* and $Q_{1}$, the influence of these parameters on the RMSEs of the proposed estimator is investigated here.

**Figure 3:**
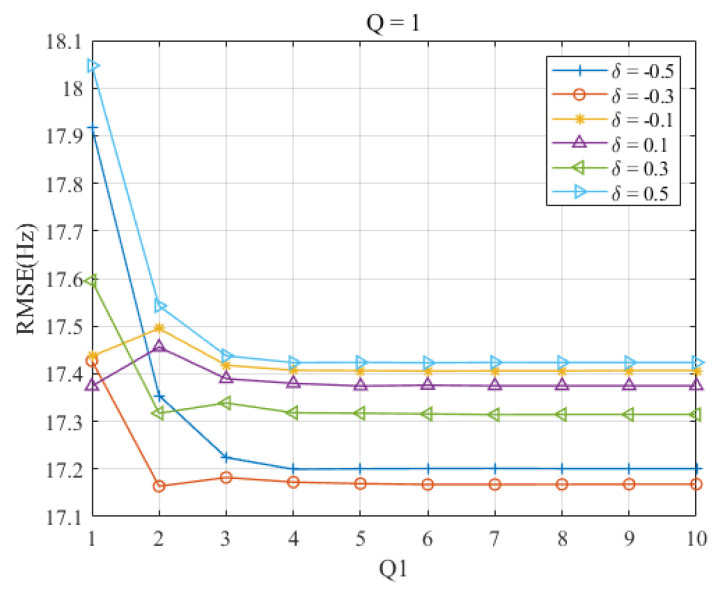
RMSEs of proposed Ds-IpDTFT estimator versus $Q_{1}$ for |δ|=0.1,0.3, and 0.5 at Q=1.

Figure 3 shows the RMSEs of the Ds-IpDTFT estimator as a function of $Q_{1}$ for each δ at Q=1. Here, RMSEs decrease as $Q_{1}$ increases when $Q_{1}\leq 3$, then remain stable, irrespective of changes in $Q_{1}$. This suggests that the dichotomous search can provide a promising estimation accuracy with $Q_{1}$ values greater than 2 at Q=1. Thus, $Q_{1}=2$ or $Q_{1}=3$ can be used as experimental parameters in Section 4.

**Figure 4:**
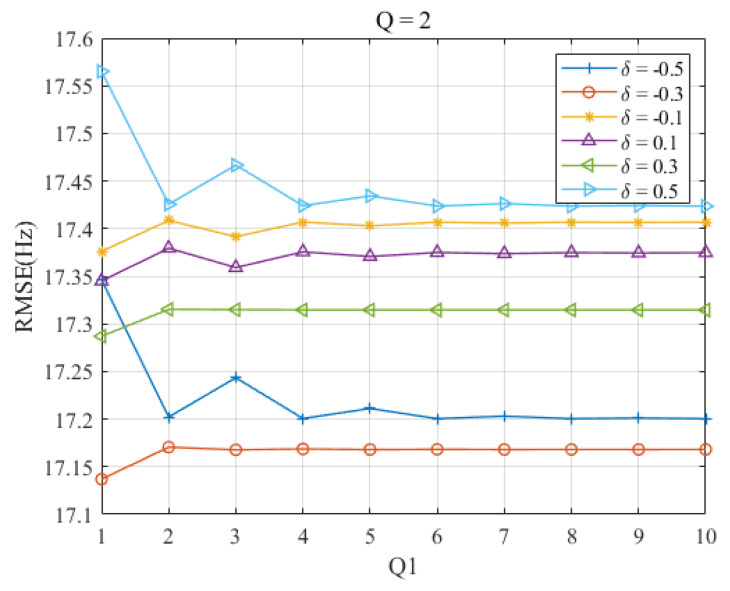
RMSEs of proposed Ds-IpDTFT estimator versus $Q_{1}$ for |δ|=0.1,0.3, and 0.5 at Q=2.

In Figure 4, the RMSEs of the proposed estimator are shown as a function of $Q_{1}$ for each δ at Q=2. Interestingly, the RMSEs decrease only when δ=±0.5 as $Q_{1}$ increases. This can be attributed to the fact that the interpolation of the generalized interpolation estimator after two iterations provides a more precise result compared with the dichotomous search, even though it requires more DTFT computation during one iteration.

Upon comparison of Figure 3 and Figure 4, it is observed that the configurations of $Q_{1}=3,$ Q=1 and $Q_{1}=2,\;Q=2$ provide the best balance between estimation accuracy and computation complexity. In fact, both configurations require almost the same computational effort for the proposed estimator. This can be deduced based on the computation complexity analysis in Section 4.

## 4. Simulations

This section presents a comparative analysis of the accuracy of several estimators, namely the proposed Ds-IpDTFT estimator, Jacobsen estimator [19], Candan estimator [36], Fan estimator [33], Aboutanio & Mulgrew (A & M) estimator [26], rational combination of three spectrum lines (RCTSL) estimator [20], and Fang estimators [25], as evidenced by experimental results. We conducted these experiments with N=512 signal samples, with a consideration of M=2N for Section 4.1. For each Monte-Carlo simulation, the initial phase, $\phi_{0}$, is randomly generated within the interval [0,2π). A sampling rate of 512 kHz is employed, and all results are gathered from 30,000 simulation runs.

### 4.1. Discussion on SNR

**Figure 5:**
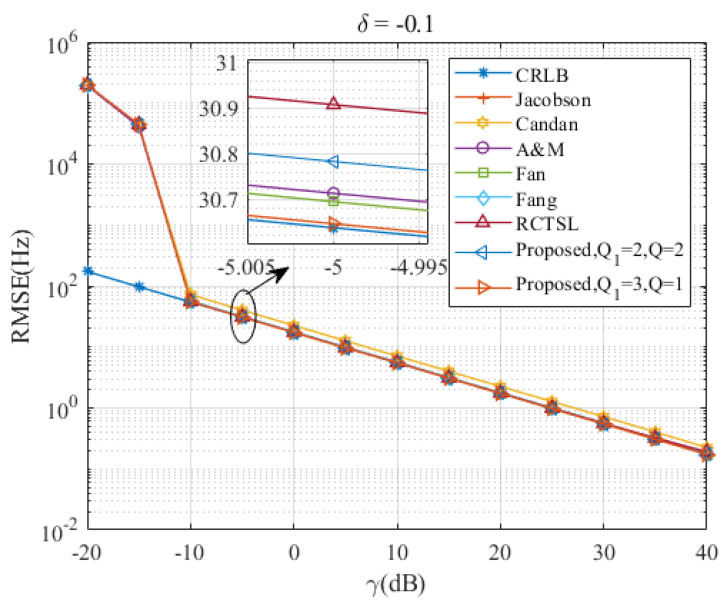
RMSEs of the five estimators versus γ at δ=−0.1.

**Figure 6:**
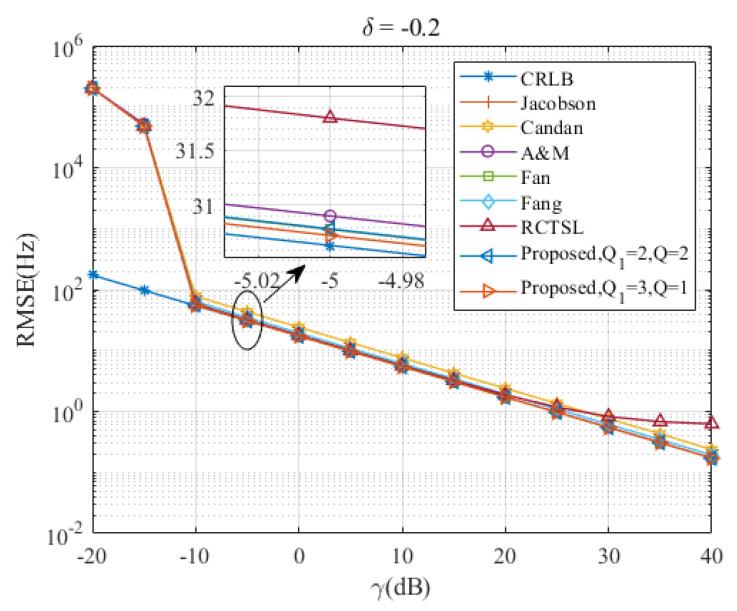
RMSEs of the five estimators versus γ at δ=−0.2.

**Figure 7:**
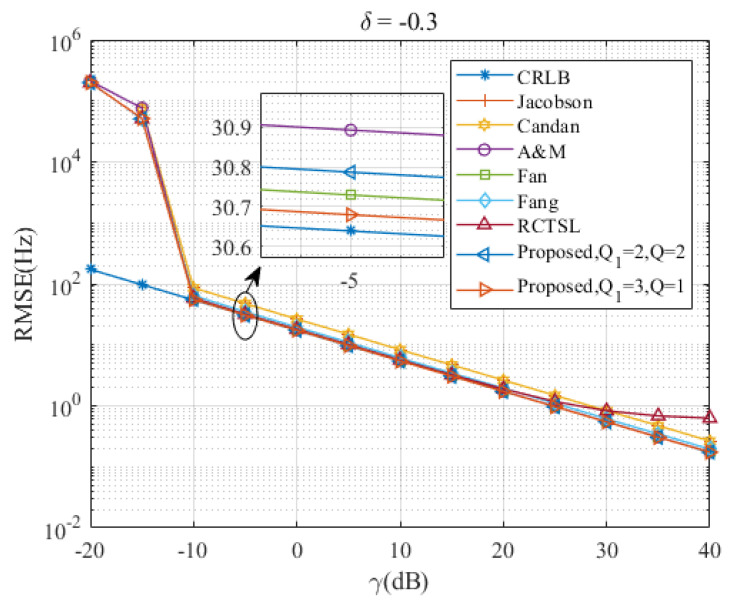
RMSEs of the five estimators versus γ at δ=−0.3.

**Figure 8:**
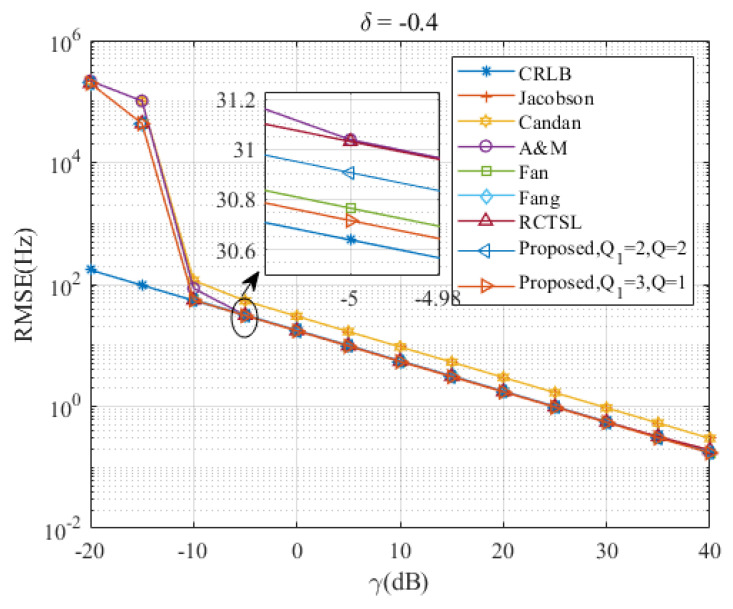
RMSEs of the five estimators versus γ at δ=−0.4.

**Figure 9:**
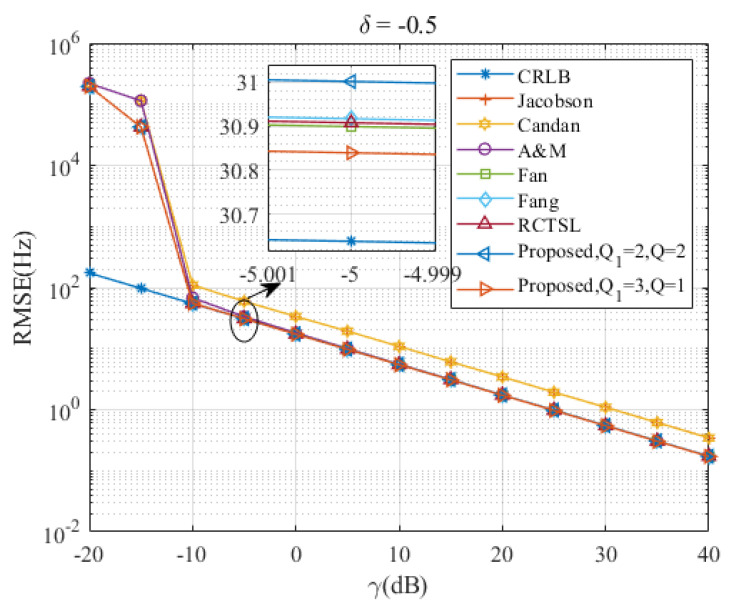
RMSEs of the five estimators versus γ at δ=−0.5.

Figure 5, Figure 6, Figure 7, Figure 8 and Figure 9 illustrate the RMSE curves of the seven estimators as functions of γ, considering a range of δ from −0.5 to −0.1. The RMSEs of estimators with comparable accuracy at γ=−5 dB are further examined for a detailed performance assessment.

Insights from these figures reveal that the SNR thresholds of these interpolation estimators hover around −10 dB, with the RMSEs of these estimators closely approximating the CRLB from γ=−10 dB to 40 dB. However, a noticeable discrepancy in the RMSEs exists between the Jacobsen estimator, Candan estimator, and the other simulated estimators, a gap that amplifies as $\left|\delta \right|$ increases. In contrast, the Ds-IpDTFT estimator’s RMSEs remain quite proximal to the CRLB across the range of δ=−0.5 to δ=−0.1. A closer look at the results for γ=−5 dB indicates that the proposed estimator with $Q_{1}=3,\;Q=1$ records the smallest RMSE among the simulated estimators for the five values of δ. Interestingly, a trend emerges where the accuracy of the A & M estimator deteriorates as δ increases. This is attributed to the A & M estimator’s high dependency on the value of δ, while the proposed Ds-IpDTFT estimator manages to minimize the influence of δ by utilizing a dichotomous search during the second estimation stage.

Additionally, some estimators show a different trend, where their RMSEs are influenced by the value of γ and deviate from the CRLB, as opposed to the Ds-IpDTFT estimator, which adheres closely to the CRLB. For instance, in Figure 6 and Figure 7, the RMSEs of the RCTSL estimator escalate rapidly when the SNR exceeds 25 dB. Similarly, Figure 8 and Figure 9 show that the RMSEs of the A & M estimator noticeably increase when the SNR is below 5 dB.

Taking into account the RMSE gaps between the Jacobsen estimator, Candan estimator, and the other simulated estimators, we decided to exclude these two estimators from the subsequent performance evaluation of the simulated estimators demonstrating comparable accuracy.

### 4.2. Discussion on δ

**Figure 10:**
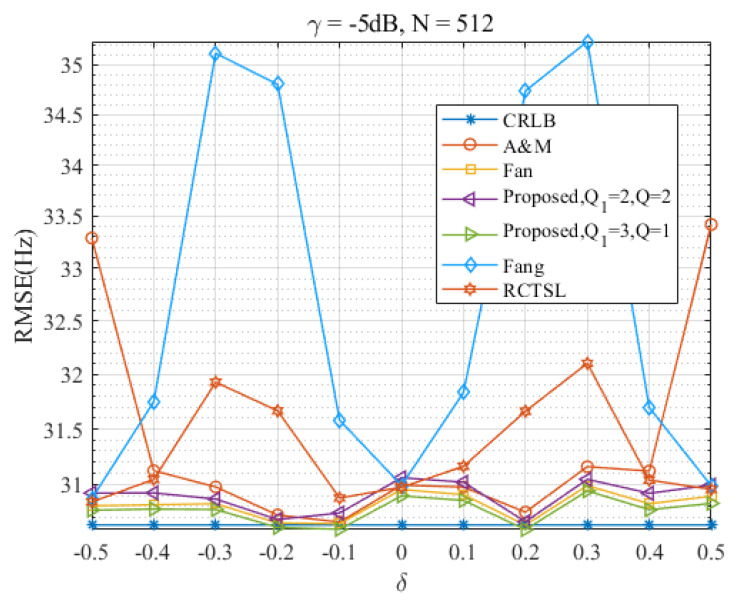
RMSEs of the four estimators versus δ at γ=−5 dB and N=512.

**Figure 11:**
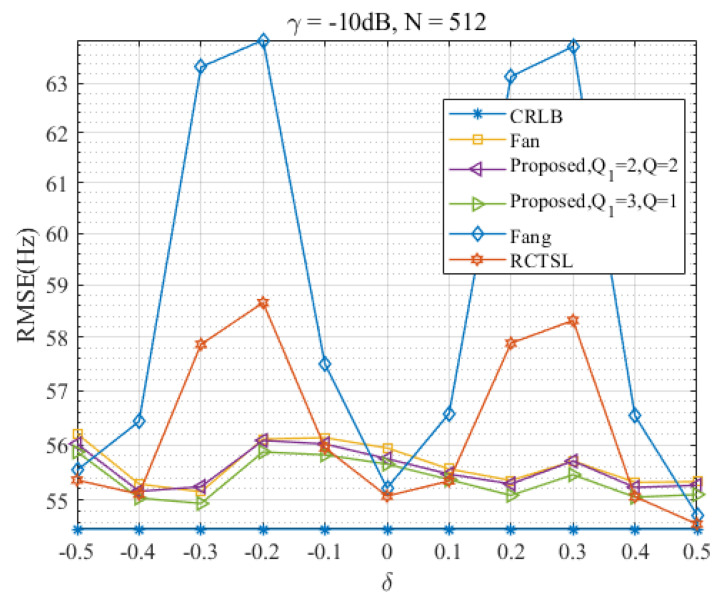
RMSEs of the five estimators versus δ at γ=−10 dB and N=512.

**Figure 12:**
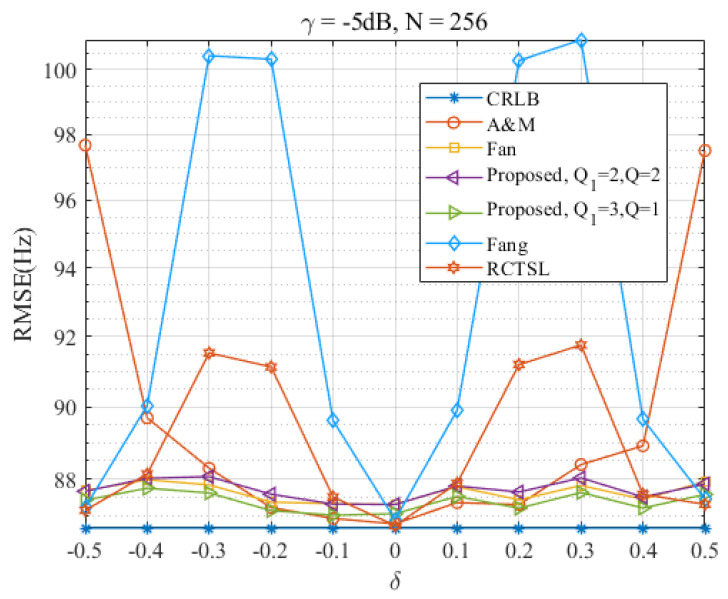
RMSEs of the four estimators versus δ at γ=−5 dB and N=256.

**Figure 13:**
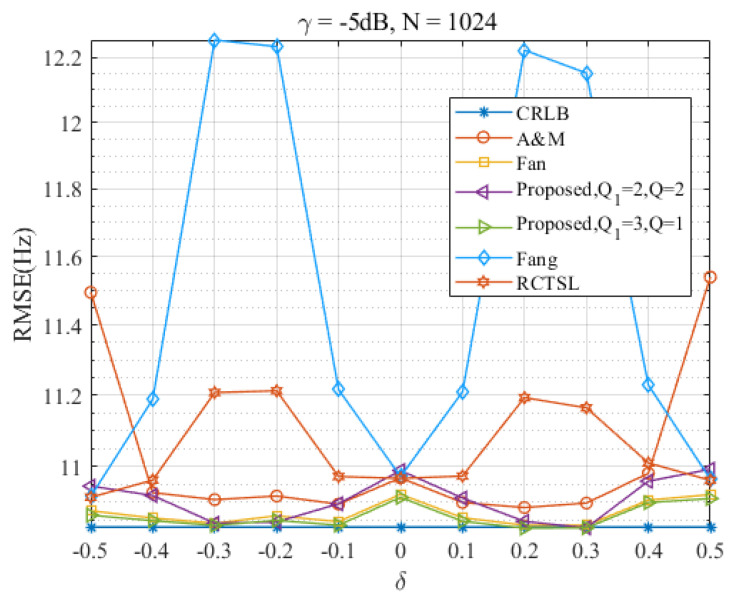
RMSEs of the four estimators versus δ at γ=−5 dB and N=1024.

In this section, we evaluate the performance of the Ds-IpDTFT estimator over the full range of δ and assess its anti-noise ability. Figure 10, Figure 11, Figure 12 and Figure 13 present RMSEs of various estimators as a function of δ, demonstrating the different estimation accuracies and robustness of these estimators.

Through Figure 10 and Figure 11, we find that the proposed Ds-IpDTFT estimator generally outperforms the other simulated estimators, with its RMSEs being consistently smaller. This superiority holds especially for $Q_{1}=3,\;Q=1$ and $Q_{1}=2,\;Q=2$, where the RMSEs hover around the CRLB across the entire δ range. Importantly, the performance of the Ds-IpDTFT estimator shows relative stability, with a small RMSE fluctuation over the estimation range. Unlike the Fang and RCTSL estimators, the Ds-IpDTFT estimator avoids an RMSE peak, a quality particularly evident around $\left|\delta \right|=0.3$.

Looking specifically at Figure 10, we note a unique advantage of the Ds-IpDTFT estimator: it avoids the edge effect around |δ|=0.5 that hampers the A & M estimator, whose RMSEs significantly increase at this point. Due to this edge effect, which intensifies as γ decreases, the A & M estimator’s RMSE curve is omitted from Figure 11 to allow a more effective comparison of the remaining estimators.

Furthermore, a comparison of Figure 10 with Figure 11 reveals the robust anti-noise performance of the Ds-IpDTFT estimator, whose RMSEs remain low even when the SNR drops.

Turning to Figure 12 and Figure 13, which show RMSE curves for γ=−5 dB at different signal lengths, N=256 and N=1024, we see that an increase in *N* leads to a reduction in the RMSE of the Ds-IpDTFT estimator for Q1=3, Q=1—dropping from 1.008 times the CRLB to 1.0026 times the CRLB. At the same time, the edge effect of the A & M estimator and the peak magnitude of the RCTSL and Fang estimators also diminish. This observation reinforces the idea that selecting a large *N* contributes to achieving better estimation accuracy, a finding consistent with the results in Figure 2.

### 4.3. Computation Complexities of Simulated Estimators

The computational complexity of common iterative interpolation estimators is primarily composed of two components. The first component originates from the DFT computation, which is primarily determined by the number of signal samples. For an *N*-point DFT, if the signal length $N=2^{l}$, the complex computation can be reduced to $\frac{N}{2}log_{2}N$ complex multiplications and $Nlog_{2}N$ complex additions through fast Fourier transform (FFT) computation. The second component consists of either four or five discrete time Fourier transform (DTFT) computations, depending on the interpolation formula and assuming the iteration number is equal to 2. Computing a one-bin DTFT of N-point samples necessitates N−1 complex additions and *N* complex multiplications. Given these considerations, we have calculated the complex computation load of the five estimators, which are presented in Table 1.

In Table 1, the additional complex multiplications and additions, which supplement those used in the FFT computation, are used to compute the DTFT samples and the residual frequency offset, $\hat{\delta }$. If three dichotomous search iterations and one interpolation iteration are adopted in the proposed estimator, three one-bin DTFT computations are required. Thus, its complex multiplication number and complex addition number are $\frac{M}{2}log_{2}M+3N$ and $Mlog_{2}M+3\left(N-1\right)$, respectively. Alternatively, if two dichotomous search iterations and two interpolation iterations are implemented, five one-bin DTFT computations are required, two from the dichotomous search and three from the second iteration of the generalized iterative interpolation estimator. In comparison with other estimators of similar estimation accuracy, the proposed Ds-IpDTFT estimator possesses a lower computational complexity. Compared with the Fan estimator, for $Q_{1}=2$ and Q=2, the complex multiplications and additions are reduced by eight times and N+1 times, respectively, and for $Q_{1}=3$ and Q=1, the complex multiplications and additions are reduced by 2N times and 2N+4 times, respectively. One complex multiplication requires four real multiplications, and one full-precision multiplication needs 13 clock periods for Xilinx V5 chips. If the clock frequency is 200 MHz, the two configurations of Ds-IpDTFT save 8×4×13×5 ns = 2080 ns and 2N×4×13×5 ns for the calculation of complex multiplications, respectively.

## 5. Experiments

**Figure 14:**
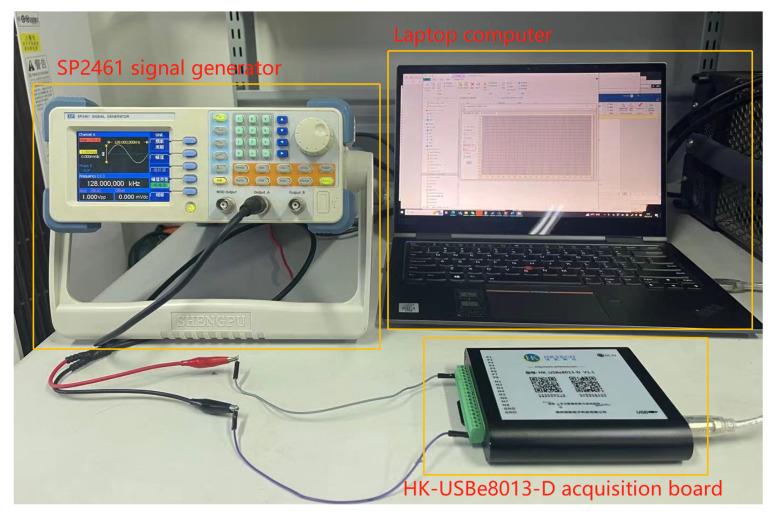
The experimental setup featuring the SP2461 signal generator, the HK-USBe8013-D acquisition board, and a laptop computer.

The performance of the proposed algorithm was validated through experiments using real-world data. A signal was generated by an SP2461 signal generator with the following parameters: *A* = 1 V and $f_{c}=\left(128+100\cdot i\right)$ kHz, where i=0,1,…,9. Signal samples were then acquired using an HK-USBe8013-D acquisition board with a sampling frequency of $f_{s}=1$ MHz. Each frequency was subjected to 5000 runs of signal samples, with each run consisting of N=1024 samples. Additionally, MATLAB simulations were carried out to mimic conditions with strong noise. A visual representation of the experimental setup for frequency estimation is provided in Figure 14.

**Figure 15:**
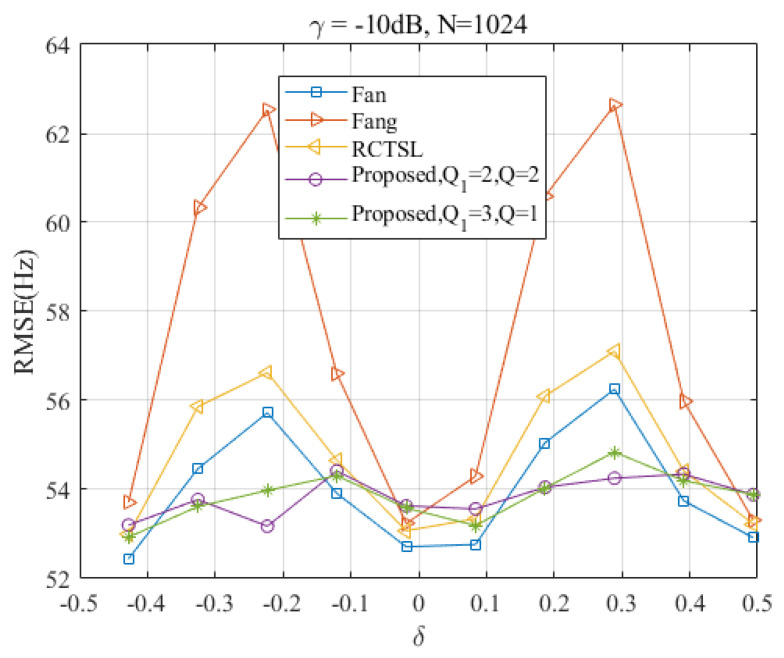
RMSEs of the four estimators versus δ at γ=−10 dB and N=1024.

To assess the performance of the proposed algorithm in comparison with other methods, Figure 15 presents the RMSEs of four different algorithms as functions of the frequency offset at γ=−10 dB and N=1024. From this plot, it can be observed that the proposed algorithm and the Fan estimator achieve a lower estimation variance in most cases when compared with the other two estimators. Moreover, the proposed algorithm maintains a stable and relatively low estimation variance across the entire estimation range. In Figure 15, the RMSEs of the three estimators for comparison show significant pulse fluctuations around δ=0.25, but the RMSE of the proposed estimator remains close to the CRLB. Although the RMSE of the Fan estimator is slightly lower than that of Ds-IpDTFT at some points, the Ds-IpDTFT exhibits a more stable performance throughout the simulation range of δ. This consistent performance echoes the simulation results discussed earlier in Section 4.

## 6. Conclusions

This research thoroughly investigates the Ds-IpDTFT estimator, a pioneering approach for fine frequency estimation of noisy exponential signals. The study illuminates the estimator’s exceptional efficiency and reduced computational complexity, as it utilizes a dichotomous search process prior to iterative interpolation estimation. This innovative design enables RMSEs to approximate the CRLB across the entire estimation spectrum, which significantly reduces computational demand.

The foundational concept of the Ds-IpDTFT estimator is rooted in the intricate exploration of the relationship between the optional parameter, *p*, and the unknown parameter, δ. While this work makes substantial strides in frequency estimation, it also acknowledges potential avenues for future research. One such avenue includes refining the selection process for parameter *p*, which could further optimize the performance of the estimator. It is a promising way to refine the selection accuracy of *p* by designing an adaptive search algorithm.

Beyond theoretical analysis, the study validates the Ds-IpDTFT estimator’s effectiveness through comprehensive simulations and real-world experiments. These empirical analyses consistently affirm the estimator’s superior performance relative to other established estimators, both in terms of estimation accuracy and computational efficiency. The Ds-IpDTFT estimator’s robustness in noisy conditions and stability across varying frequencies is particularly noteworthy, marking it as a promising tool for practical applications in signal processing and related fields. For high-speed mobile terminals and satellite terminals with limited hardware resources, the proposed estimator provides a reliable and fast carrier estimation method. Specifically, it can be utilized for parameter estimation and synchronization in wireless communications, radar systems, power systems, mechanical vibration analysis, and biomedical signal processing. As computing hardware continues to advance, the computational complexity constraints of DFT will be gradually alleviated, further expanding the applicability of the proposed methodology.

## Figures and Tables

**Table 1:** Computational requirements for the simulated estimators.

Estimators	Complex Multiplications	Complex Additions
Fang (*M* points)	$\frac{M}{2}log_{2}M$	$Mlog_{2}M$
RCSTL (*M* points)	$\frac{M}{2}log_{2}M+1$	$Mlog_{2}M$
A & M (*N* points, 2 iterations)	$\frac{N}{2}log_{2}N+4N+2$	$Nlog_{2}N+4N$
Fan (*M* points, 2 iterations)	$\frac{M}{2}log_{2}M+5N+8$	$Mlog_{2}M+5N+1$
Proposed (*M* points, 2 dichotomous searches, 2 iterations)	$\frac{M}{2}log_{2}M+5N$	$Mlog_{2}M+5\left(N-1\right)$
Proposed (*M* points, 3 dichotomous searches, 1 iteration)	$\frac{M}{2}log_{2}M+3N$	$Mlog_{2}M+3\left(N-1\right)$

## Data Availability

Not applicable.
